# Adipose tissue plasticity: how fat depots respond differently to pathophysiological cues

**DOI:** 10.1007/s00125-016-3933-4

**Published:** 2016-04-04

**Authors:** Vanessa Pellegrinelli, Stefania Carobbio, Antonio Vidal-Puig

**Affiliations:** University of Cambridge Metabolic Research Laboratories, Level 4, Wellcome Trust-MRC Institute of Metabolic Science, Box 289, Addenbrooke’s Hospital, Cambridge, CB2 OQQ UK; Wellcome Trust Sanger Institute, Wellcome Trust Genome Campus, Hinxton, Cambridge, UK

**Keywords:** Adipogenesis, Adipose tissue, Development, Fibrosis, Inflammation, Obesity, Plasticity, Review, Tissue remodelling, Type 2 diabetes

## Abstract

White adipose tissue (WAT) has key metabolic and endocrine functions and plays a role in regulating energy homeostasis and insulin sensitivity. WAT is characterised by its capacity to adapt and expand in response to surplus energy through processes of adipocyte hypertrophy and/or recruitment and proliferation of precursor cells in combination with vascular and extracellular matrix remodelling. However, in the context of sustained obesity, WAT undergoes fibro-inflammation, which compromises its functionality, contributing to increased risk of type 2 diabetes and cardiovascular diseases. Conversely, brown adipose tissue (BAT) and browning of WAT represent potential therapeutic approaches, since dysfunctional white adipocyte-induced lipid overspill can be halted by BAT/browning-mediated oxidative anti-lipotoxic effects. Better understanding of the cellular and molecular pathophysiological mechanisms regulating adipocyte size, number and depot-dependent expansion has become a focus of interest over recent decades. Here, we summarise the mechanisms contributing to adipose tissue (AT) plasticity and function including characteristics and cellular complexity of the various adipose depots and we discuss recent insights into AT origins, identification of adipose precursors, pathophysiological regulation of adipogenesis and its relation to WAT/BAT expandability in obesity and its associated comorbidities.

## Introduction

Obesity and its metabolic complications (e.g. type 2 diabetes, cardiometabolic disorders) contributing to the metabolic syndrome represent one of the most important public health problems, with societal and economic implications urging for new therapeutic strategies and effective social policies. White adipose tissue (WAT) plays a key homeostatic role, not only by ensuring efficient energy storage but also by its quick mobilisation (lipids) to ensure peripheral demands. WAT is highly vascularised and innervated as would be expected from a sophisticated constituent of a hormonal homeostatic system [1]. To be able to accommodate the excess energy during the course of obesity, WAT undergoes various cellular and structural remodelling processes: (1) tissue expansion through coordination of increased adipocyte size (hypertrophy) and/or number (hyperplasia) [2]; (2) recruitment of inflammatory cells [3] and (3) remodelling of the vasculature and the extracellular matrix (ECM) to allow adequate tissue expansion, oxygenation and mobilisation of nutrients [4, 5]. However, when obesity and inflammation are sustained, these adaptive homeostatic mechanisms fail, leading to WAT dysfunction characterised by impaired secretion of adipokines, abnormal lipid storage and adipogenesis, exacerbated fibrosis deposition and insulin resistance.

WAT is organised in discrete anatomical depots identified as subcutaneous adipose tissue (SAT) and visceral adipose tissue (VAT); the expansion of SAT and VAT contributes to obesity and related complications [6]. The ‘adipose tissue expandability model’ identifies the limited capacity and dysfunctionality of WAT, preventing its expansion and accommodation of surplus of energy, as key determinants for the onset and progression of obesity-associated metabolopathologies as a result of ectopic deposition of toxic lipid species in metabolic organs (i.e. muscle or liver [also known as lipotoxic insult]) [7]. Appropriate WAT plasticity and expandability seem to guard against metabolic disorders [7]. Moreover, promotion of SAT expansion to act as a buffer of lipids is a strategy that may limit the deleterious metabolic effects of VAT [8]. Following a similar concept, transplantation of SAT or removal of VAT in obese mice reversed adverse metabolic effects of obesity and improved glucose homeostasis [9, 10].

There is also evidence that the deleterious effects mediated by dysfunctional white adipocyte-induced lipid overspill can be halted by the pro-oxidative anti-lipotoxic effects mediated by brown adipose tissue (BAT) activation. The sympathetic nervous system regulates this function through β-adrenergic stimulation of brown mature adipocytes’ dissipation of energy in the form of heat mediated by mitochondrial uncoupling protein-1 (UCP-1) activation. UCP-1-expressing multilocular adipocytes, termed ‘beige’ or ‘brite’ (brown-in-white) adipocytes, can also be found interspersed among white adipocytes within SAT under conditions requiring increased heat production (e.g. chronic cold exposure). Increasing BAT/beige mass has been suggested as a potential therapeutic approach to treat human obesity/diabetes supported by recent studies reporting that, like rodents, humans display highly metabolically active BAT [11–13]. BAT atrophy is observed in obese individuals in association with increased visceral fat, ageing and hyperglycaemia [11], suggesting that defective BAT may exacerbate the development of obesity/complications. However, it cannot be discarded that fat-mediated thermo-insulation may have contributed to BAT regression in these patients.

Departing from the previous evidence, two therapeutic strategies have been tested: (1) improving adipose tissue (AT) plasticity either by expanding anabolic functions of white adipocytes and/or (2) increasing tissue thermogenesis through activation of pre-existing brown adipocytes and/or recruitment and differentiation of brown pre-adipocyte precursors [14]. The success of these strategies may be limited by the uncertainty regarding the identity and origins of adipocytes from different depots and the limited information available about how obesity-associated changes in cellularity/fibro-inflammation influence WAT plasticity. Thus a better understanding of the molecular mechanisms and cellular mediators that control AT plasticity and expansion is essential.

In this review, we discuss the current understanding of the origins of WAT, the identity of white/brown/brite adipocyte progenitors (APs) and how depot-specific vascularisation and fibro-inflammation interact with adipogenesis/cell hypertrophy, including the recent insights highlighted by lineage-tracing studies in mice and genetic/genomics data obtained from humans. We will notably highlight the structural/cellular differences in humans compared with rodent models. Finally we discuss BAT plasticity and how obesity-associated environmental cues can be targeted to improve tissue activation and global metabolic homeostasis.

## Structural features involved in remodelling of the AT depots

In addition to the metabolic/functional differences reported in numerous studies [1, 15–17], the SAT, VAT and BAT depots also exhibit differences at cellular and structural levels that may have an impact on tissue plasticity and remodelling (Table 1). For instance, lean individuals display larger adipocytes in SAT than in VAT whereas mouse studies have shown the presence of smaller adipocytes in SAT than in retroperitoneal VAT [1]. While this discrepancy between the two species has yet to be elucidated, cellular heterogeneity in terms of adipocyte size is also present among human SAT depots depending on body distribution and functional and structural characteristics (more specifically the ECM properties) [18]. In the context of obesity the fibrous ECM may become a limiting factor for adipocyte size (discussed later in the review). BAT differs from other fat pads at the morphological/molecular level (i.e. vascularisation, innervation) and also by virtue of its unique thermogenic capacity [14] (Table 1). This depot is the dominant site of non-shivering thermogenesis in rodents and is also highly present in infants, maintaining body temperature and warming the blood flow of key organs. Of note, BAT depots persist in human adults, preferentially located in cervical, supraclavicular, mediastinal, paravertebral, suprarenal and peri-renal areas [14]. Recent reports have highlighted structural differences between rodents and humans, where BAT deposits are described as being composed of adipocytes displaying a phenotype more similar to rodent beige/brite cells than to canonical brown fat [19].

**Table 1:** Structural and cellular variables involved in AT remodelling: comparison between VAT, SAT and BAT

Variable	SAT vs VAT	BAT vs WAT
Adipogenic potential		
Adipogenic genes	Higher expression of *Cepbá*, *Pparγ* (*Pparg*), *Dkk2*, *Stat5* (*Stat5a*), *Bmp2*, *Bmp4* [71]^a,d^	Lower differentiation potential [106]^a,d^
Anti-adipogenic genes	Lower expression of *Gata2*, *Tgfb2* and *Pparγ* [71]^a,d^	Higher *Pparg2* mRNA expression (lean/obese) [107–109]^a,c^
MSC markers	Lower expression of *Lif*, *Igf1*, *Igfbp7*, *Ctgf*, *Mgp*, *Trib2*, *Pgn1*/*Bgn* [71]^a,d^	Lower plasticity, mesenchymal stem cells [106]^a,d^
Vascularisation		
Total vascular density	Lower compared with oVAT (obese) [110]^b,c^	Higher [111]
Capillary density	Higher than oVAT of (obese) [112]^b,c^	Greater [111]^a,c^, 3 capillaries per adipocytes in BAT compared with 1 per adipocytes in WAT [113]^a,c^
Vascular sprouting	Greater [112]^b,c^	NA
Innervation		
Neurogenic factors	Lower mRNA expression of *Nnat* and *Nrg4* than gVAT [114]^a,c^	Lower mRNA expression of *Nnat* than gVAT but not *Nrg4* [114]^a,c^
Nervous network	NA	Greater number of noradrenergic parenchymal nerve fibres [115]^a,c^
Cellularity		
Immune cells	Higher CD68^+^ cells (obese adolescent) [109], but lower compared with m/oVAT of lean [116] and obese [117] individuals^b,c^	Lower haematopoietic population (CD45^+^) [106]^a,c^
SVF (except APs)	Higher [71]^a,d^	Lower F4/80-, CD68- and CD11b^+^ cells compared with iSAT/eVAT (lean/obese) [92, 118]^a,c^
Adipocyte death/CLS	Lower [78, 117]^a,b,c^	NA
ECM		
Tissue expression	Greater protein expression of type 1 collagen but lower level of laminin (b/c) and fibronectin (lean) [69]^a,c^	NA
	Higher *COL6A3* mRNA expression (lean/obese) [119]^b,c^	
Secretion	Higher secretion of THSB1/2, type 1 collagen, SPARC, TIMP1. Lower secretion of laminin, type 6 collagen and TGFβ1 [120]^b,c^	NA

Comparative studies below were performed in WAT/BAT tissues from lean and/or obese rodents and humans or isolated SVF cells from various depots: SAT (inguinal, iSAT in rodents) and VAT (gonadal [gVAT], epididymal [eVAT] in rodents; omental [oVAT] in humans)

^a^Rodents

^b^Humans

^c^WAT/BAT tissues

^d^Isolated SVF cells

CLS, crown-like structure; NA, not available; SPARC, secreted protein acidic and cysteine rich; THSB1/2 thrombospondin-1/-2; TIMP1, tissue inhibitor of metalloproteinase 1

## AT progenitors and development

In humans, WAT forms during the second trimester of pregnancy [20] and develops (like in other species) in an anterior to posterior, rostral to caudal and dorsal to ventral direction [21]. The recently developed ‘AdipoChaser’ mouse model [2] precisely elucidated the SAT/VAT developmental timing in mice enabling temporally controlled detection of mature adipocytes and identification of newly formed adipocytes. This model revealed that SAT adipocyte commitment and differentiation occurs early during embryogenesis, in E14–E18, in both sexes and that the number of adipocytes remains very stable postnatally. In contrast, epididymal adipocytes preferentially differentiate postnatally. This process occurs gradually over a relatively long period of time, after birth recruitment of brown-like-adipocytes in SAT has occurred at approximately P10, at room temperature, and disappears spontaneously at around P30. Interestingly, these cells can re-emerge in response to cold or to treatment with a β_3_-adrenergic agonist [22].

With respect to BAT development, lineage-tracing studies using Engrailed-1 (*En1*)-CreERT-inducible mice crossed with Rosa-floxed Stop-*LacZ* mouse, revealed that E14.5 is the stage at which BAT becomes visible in mouse embryos [23]. However, the divergence between myoblast and BAT precursors already occurs between stage E9.5 and 11.5 in mice [24]. In humans, BAT is detectable at birth, in early childhood and also in adult individuals [11, 12], but the exact embryonic stage at which it makes its first appearance is still unknown.

### Embryonic origins of adipocytes

Lineage-tracing studies have shown that brown adipocytes and myocytes share common myogenic factor 5 (MYF5)^+^, paired box 3 (PAX3)^+^ and paired box 7 (PAX7)^+^ progenitors that originate in the paraxial mesoderm [14, 25]. Given the absence of this myogenic signature in white adipocytes and their progenitors, it was concluded that white adipocytes would originate preferentially from MYF5^–^ precursors. This assertion was recently challenged by a study in which the conditional deletion of *Pten* driven by *Myf5*-Cre caused an overgrowth of BAT and also a paradoxical overgrowth of specific WAT pads and the loss of others [26]. Subsequent lineage-tracing studies have confirmed the presence of some MYF5^+^ and PAX3^+^ adipocyte progenitors in WAT, indicating that white APs can derive from both MYF5^+^/PAX3^+^ and MYF5^–^/PAX3^+^. Following on from these studies, the adipocyte origin from MYF5^−^/PAX3^−^ lineages is still not clear.

In addition to a mesodermal origin for adipocytes, the neural crest (NC) also seems to give rise to a subset of adipocytes localised in the salivary gland and ears. An in vivo lineage-tracing approach using a *Sox10*-Cre/Rosa26-YFP model, where NC-derived cells are constitutively labelled, has provided evidence for the contribution of the NC to the adipocyte lineage during normal development [27]. Similarly, another cell fate mapping strategy in mice showed that the earliest wave of mesenchymal stem cells (MSCs) in the embryo is generated from sex-determining region Y-Box 1 (SOX1)^+^ neuroepithelium, in part through a NC intermediate stage [28].

### Adipocyte progenitors in adult AT

Determination of cell surface markers of APs has been a priority in most studies attempting to elucidate the developmental origin of the adipocyte lineage and identify distinct cellular intermediates between MSCs and mature adipocytes. FACS technology has been used extensively to isolate cell subpopulations from WAT stromal vascular fraction (SVF) based on various cell surface markers, which were then tested for their adipogenic potential in vitro and in vivo after transplantation in lipoatrophic A-Zip mice [29]. Following this strategy, APs were identified as Lin^−^/CD34^+^/CD29^+^/Sca-1^+^/CD24^+^ cells able to form white fat when subcutaneously transplanted.

Recent investigations of the close temporal–spatial association between angiogenesis and adipogenesis suggested that the adipose niche is located adjacent to the growing vasculature and that adipocytes may have endothelial origins. In particular, lineage-tracing studies using the endothelial marker VE-cadherin or the pre-adipocyte marker zinc finger protein 423 (ZFP423) also suggested that some brown and white adipocytes could originate from endothelial progenitors [30, 31]. Similarly, Shan et al identified aP2-expressing progenitors in SVF of both WAT and BAT [32]. In a more recent study, perilipin^+^/adiponectin^+^ pre-adipocytes were found to emerge at embryonic day 16.5 in WAT and proliferated to form clusters interacting with growing adipose vasculature until birth while co-expressing stem cell markers such as cluster of differentiation (CD)24, CD29 and platelet-derived growth factor receptor α (PDGFRα) [33]. Some pre-adipocytes derived from PDGFRβ^+^ mural cells. This study indicates that the endothelial origin of adipocytes is also embryonal. However, the view of an endothelial source of adipocytes is challenged by some lineage-tracing studies using other endothelial markers (i.e. cadherin-5 [Cdh5] and tyrosine kinase with immunoglobulin-like and EGF-like domain 2). Cdh5-expressing cells were traced by using *Cdh5*-Cre:*mT/mG* and failed to show any Cdh5-derived adipocyte precursors within the SVF. Analysis performed with receptor tyrosine kinase *Tie2-*Cre produced similar negative results [34].

### Specific origins for VAT and SAT

One major gap in adipocyte biology is the incomplete understanding of the developmental origins of WAT. A recent study showed that VAT but not SAT arises from cells expressing the Wilms’ tumour 1 gene (*Wt1*) late in mouse gestation [35]. *Wt1* continues then to be expressed in VAT progenitors into adult life. The authors of this study also showed that VAT is lined by mesothelium and provided evidence that this structure is the source of adipocytes. Conversely, another study showed that the majority of the precursor and mature subcutaneous white adipocytes in adult C57Bl/6 mice are labelled by *Prx1*-Cre whereas few to no brown adipocytes or visceral white adipocytes are labelled [36].

### In between white and brown: origins of beige adipocytes

Considering that beige adipocytes can arise from white APs by in vitro chronic exposure to peroxisome proliferator-activated receptor γ (PPARγ) agonists, it is likely that they may share the same origin as most white adipocytes [37]. Recent evidence from adults suggests that beige adipocytes may form either by interconversion from white adipocytes or by proliferation and differentiation from specific precursors [2, 38]. Using mice in which UCP-1-expressing cells are constitutively or transiently labelled with fluorescent markers, beige adipocytes recruited after cold exposure were found to originate directly from white adipocytes. Although cold exposure has not yet been proven to induce SAT browning in humans [39], a recent study showed the presence of beige adipocytes in SAT of burns victims [40]. This is probably due to the chronically elevated circulating levels of noradrenaline (norepinephrine) found in their blood as part of a severe adrenergic stress response. Progressive recruitment of UCP-1^+^ multilocular adipocytes was observed in serial SAT biopsies obtained from these patients, possibly resulting from transdifferentiation of mature white cells.

Results supporting the ‘specific precursor hypothesis’ come from a study using the ‘AdipoChaser model’ indicating that cold-recruited beige cells are produced by clonal expansion of a precursor cell [2]. This is consistent with reports that beige adipocytes arise de novo in WAT in response to adrenergic stimulation as indicated by tracking beige adipogenesis using BrdU accumulation [41]. Similar lineage-tracking approaches identified self-renewing PDGFRα^+^ precursors as a significant source of newly formed beige adipocytes; these PDGFRα^+^ progenitors are ‘bi-potential’, having the ability to produce both beige and white adipocytes when cultured in vitro [41]. In humans, native CD45^−^/CD34^+^/CD31^−^ cells were identified initially as human white APs [42, 43]. However, when additionally selected for the cell surface marker MSC antigen 1 they showed potential to become both white and beige in response to specific stimuli [44]. Beige adipocytes may also derive from dedicated beige adipocyte precursors, as indicated by a study characterising the in vitro adipogenic potential of immortalised WAT- and BAT-derived precursors showing that some of the WAT precursors differentiated preferentially into beige adipocytes [19]. This suggests the existence of different types of adipocyte precursors in WAT, differing in their potential to produce beige adipocytes perhaps due to their lineage origins. For example, PAX3^−^ or MYF5^–^ adipocyte precursors isolated from WAT possess a higher potential to differentiate into brown-like cell genes compared with PAX3^+^ or MYF5^+^ precursors, respectively [45, 46].

In adult humans, inducible ectopic brown-like/beige depots have been observed in WAT surrounding the adrenal gland when the medulla develops a catecholamine-secreting tumour (i.e. phaeochromocytoma) [47]. Brown adipose stem cells were isolated from this peri-adrenal fat depot, which expresses brite/classical BAT markers and high levels of UCP-1, and their properties were compared with those of SAT precursors from the same patients. The findings demonstrated that BAT developing in peri-adrenal WAT derives from adult stem cells, unlike WAT precursors, suggesting an independent origin of the two fat depots.

**Table:** 

SAT and BAT adipogenesis occurs during embryogenesis while VAT adipocytes preferentially differentiate postnatally. While BAT originates from paraxial mesoderm, WAT can have mesodermal and NC origins. White/brown adipogenesis can be reinitiated in adults in response to positive energy balance by differentiation of APs located within the vasculature. Whether the origin of a third class of adipocytes, ‘beige/brite’ adipocytes, is the result of white adipocyte trans-differentiation or differentiation of specific precursors is still a matter of debate	

## Molecular and structural factors regulating adipogenesis

### Adipogenic cascade and molecular regulation

**Fig. 1:**
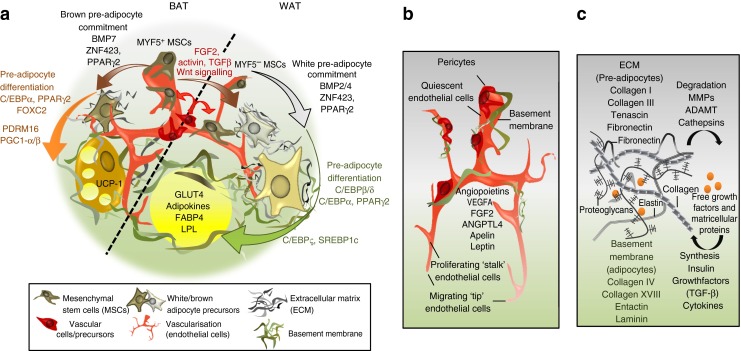
BAT/WAT adipogenesis and associated tissue remodelling. (**a**) Adipogenesis consists of a two-step process involving, successively, mesenchymal precursors, committed pre-adipocytes, growth-arrested pre-adipocytes, mitotic clonal expansion, terminal differentiation and mature adipocytes. The first step of white adipocyte differentiation is the generation of pre-adipocytes from mesenchymal precursors (MSCs) MYF5^−^ (grey arrow) or MYF5^+^ (brown arrows), driven by BMP4. By promoting dissociation of the WISP2–ZNF423 complex, BMP4 allows nuclear entry of ZNF423 and PPARγ induction. Repression of ZNF521, which negatively regulates ZNF423 by repressing EBF1, also constitutes an early event in induction of white adipogenesis. The second step of adipogenesis is the differentiation of pre-adipocytes into mature adipocytes (green arrow), a process that involves the activation of transcription factors C/EBPβ and C/EBPδ, first during mitotic clonal expansion of pre-adipocytes and subsequently induction of C/EBPα and PPARγ2, which maintains the terminal differentiation of the adipocyte. Finally, SREBP1 is considered to be the third key transcription factor for adipogenesis, inducing expression of adipocyte-specific genes such as *FABP4*, adiponectin, *GLUT4* (also known as *SLC2A4*), and *LPL*. C/EBPζ, a dominant inhibitor of C/EBPα and β, is induced in late adipocyte differentiation and has been proposed as an inhibitor of adipogenesis. Both canonical and non-canonical Wnt signalling pathways negatively regulate adipogenesis. β-Catenin mediates canonical Wnt signalling by activating cyclin D1, conversely with inhibition of PPARγ and C/EBPα, causing a further decrease in adipogenesis. Similar to WAT, commitment of brown pre-adipocytes from MYF5^+^ MSCs (brown arrows) and differentiation into brown adipocytes (orange arrow) involves transcriptional control by C/EBPs and PPARγ2 while some transcription factors, such as FOXC2, PGC1α and PDRM16, are specific to brown cell fate leading to brown-specific thermogenic markers such as UCP-1. Recent evidence suggests that both brown and white adipocytes may derive from endothelial precursors (red arrows). (**b**) Angiogenesis is driven by angiogenic factors produced by adipocytes and vascular cells. VEGF-A is considered the main pro-angiogenic factor of AT. VEGF-A binds to VEGF receptors 1 and 2 to drive the migration of so-called ‘tip cells’, the ECs at the tip of a new capillary. Other growth factors such as ANGPTL4 and FGF-2 drive the migration and proliferation of stalk cells, the endothelial cells between the tip cells and the existing vessel that drive elongation. The new vessel is stabilised by the production of ECM components, forming the basement membrane, and the recruitment of pericytes. (**c**) Pre-adipocytes are surrounded by a fibrous ECM enriched in collagen I, collagen III and fibronectin replaced by the basement membrane, a specialised ECM surrounding mature adipocytes composed of collagen IV, collagen XVIII, entactin and laminin. ECM remodelling during adipogenesis involves degradation of pre-adipocyte ECM by proteases (MMPs, ADAMT and cathepsins). This liberates growth factors and matricellular proteins that are important for the synthesis of the new mature adipocyte basement membrane. ADAMT, a disintegrin and MMP with thrombospondin motifs; ANGPTL4, angiopoietin-like 4; EBF1, early B-cell factor 1; LPL, lipoprotein lipase

White and brown adipogenesis are complex processes requiring coordination of multiple regulatory and signalling pathways. One family of proteins that contributes to the commitment of precursor cells (i.e. MSCs) to the white adipocyte programme is represented by the bone morphogenetic proteins (BMPS). While BMP4 induces differentiation of progenitor cells to white adipocytes in both humans [48] and mice [49], BMP2 does this in mice only [49]. Conversely, other factors such as fibroblast growth factor 2 (FGF-2) and activin A maintain MSCs in an undifferentiated proliferating state. White adipogenesis is also characterised by cell cycle arrest and the induction of mature white adipocyte machinery involving three key transcription factors: PPARγ2 [50], CCAT/enhancer-binding proteins (C/EBPs) and sterol regulatory element binding protein 1 (SREBP1). This has been extensively described in the literature [14] and is summarised in Fig. 1a.

As for white fat, brown adipocytes also need the induction of PPARγ2 and C/EBPα to reach their terminal differentiation. However, differentiation of brown adipocytes requires the presence of BMP7. Interestingly, BMP7 alone can stimulate the differentiation of brown pre-adipocytes and commit mesenchymal precursors to a brown adipocyte cell fate in mice [51]. BMP7 upregulates brown fat-specific markers, such as UCP-1, PRDM16 and PGC-1α/β, inhibiting the expression of anti-adipogenic molecules, such as PREF-1, WNT10a or nectin. A similar role has been described for BMP6 in both mice and humans [52]. Moreover, BMP7 and BMP8b are known to act as sensitisers of adrenergic signalling in mature brown adipocytes, leading to an increase in the sympathetic tone [53]. There are also other factors that direct the process toward a brown vs white adipocyte cell fate. For example, forkhead box C2 (FOXC2) modulates the expression and activity of adrenergic signalling molecules and PGC1α coordinates expression of both mitochondrial and thermogenic genes [54].

Concerning beige adipocytes, regardless of whether they are derived from transdifferentiation of white adipocytes or from specific precursors [41, 55], their commitment towards a brown-like phenotype is promoted in vivo and in vitro by cold stimulation and/or β_3_ agonist or T3 similarly to brown cells. The exception is that beige adipogenesis does not require C/EBPα [56]. Furthermore, a study performed in humans recently showed that, in addition to regulate white adipogenesis, BMP4 promotes the induction of a beige phenotype [57]. Other members of the BMP family have been reported to promote WAT browning in mice (i.e. BMP7 and BMP9) and humans (i.e. BMP7) [58, 59].

Some adipokines and adipocytokines such as adiponectin [60] are described to regulate BAT adipogenesis. Others such as IL-6 seem to be required for cold-induced UCP-1 expression in SAT [61]. Moreover, apelin promotes differentiation of brown adipocytes and browning of white fat by interacting with the APJ (apelin) receptor, which activates phosphoinositide 3-kinase (PI3K)/Akt and AMP-activated protein kinase signalling [62] while suppressing white adipogenesis [63].

### Vascular and ECM remodelling in adipogenesis

Appropriate vascularisation is required to ensure the development and growth of AT. Concomitantly with adipogenesis, angiogenic factors, such as FGF-2, vascular endothelial growth factor (VEGF) and human growth factor, are produced, mostly by APs, inducing a robust angiogenic response. AT growth requires an interaction between endothelial cells (ECs) and pre-adipocytes guiding cell migration via FGF- and VEGF-dependent pathways. The newly formed vessel is finally stabilised by the production of ECM and the recruitment of pericytes [4] (Fig. 1b). Overexpression of a dominant-negative form of PPARγ or the blockade of VEGF receptor 2 signalling by neutralising antibodies inhibits adipogenesis through impairment of both AT growth and angiogenesis. Conversely, pro-adipogenic factors such as PPARγ activation also promotes angiogenesis and EC motility and boosts expression levels of VEGF, VEGF-B, angiopoietin-like factor-4 [64] and BMPs, which promote endothelial specification and subsequent venous differentiation during embryonic development [65].

During adipogenesis, formation and expansion of the lipid droplet requires a morphological change of the fibroblastic pre-adipocyte involving remodelling of both actin cytoskeleton [66] and ECM (Fig. 1c). This process requires enzymes such as metalloproteases (MMPs) that catalyse the degradation of collagen. Deficiency in MMP 9/10/12 does not affect adipogenesis, whereas single allele deficiency of MMP14/2 impairs it. Moreover, knockout mice for *Mmp3*/*11*/*19*, fed a high-fat diet (HFD) display marked hypertrophy of AT [67, 68]. Given that several growth/angiogenic factors such as VEGF are sequestered in the ECM, MMPs also seem to control pre-adipocyte differentiation and microvessel maturation by regulating degradation of the ECM.

Insulin also contributes to ECM turnover through regulation of the expression of enzymes involved in the post-transductional modification of some proteoglycans such as sulfatase-2. Moreover, insulin acts at a post-transcriptional level to increase production of mature type I collagen, collagen V fragment and C-terminal peptides of type I, II and III collagen. Insulin also increases the expression of prolyl-4-hydroxylase, involved in collagen stabilisation. Finally, *COL6A2* and *TSP1* have been identified as PPARγ target genes. Of note, the composition of ECM and its evolution during adipogenesis differs among fat depots. For example, expression levels of collagen IV and fibronectin are higher in VAT than in SAT, while in contrast, SAT is highly enriched in type I collagen [69] (Table 1).

### Differences in adipocyte precursor pool and adipogenesis in AT depots

Anatomical localisation influences adipogenesis in both humans and rodents with respect to proliferation and differentiation of SVF or APs. SVF cells isolated from human and rodent SAT display greater differentiation capacity compared with those from VAT (Table 1). This has been linked to higher gene expression of regulators of adipogenesis such as CEBPα or fatty acid binding protein 4 (FABP4), as well as greater response to PPARγ agonists, thiazolidinediones (TZDs). Consistently, TZD treatment enhanced fat storage preferentially in SAT [70]. Similarly to SVF cells, APs from SAT display higher expression levels of pro-adipogenic genes (*PPARγ* [*PPARG*], *CEBPΑ*, *BMP2*, *BMP4* and *DKK2*) and differentiate better than those from VAT depot in response to classical adipogenic stimulus, the VAT requiring additional adipogenic factors such as BMP2/4 [71]. This might be partly explained by intrinsic differences of VAT APs exhibiting a ‘mesenchymal stem cell’-like phenotype with higher expression of MSC markers (leukaemia inhibitory factor, connective tissue growth factor and matrix Gla protein) and adipogenic inhibitors such as GATA-binding protein 2 and TGFB2. Finally, clonogenic assays and in vivo BrdU studies in adult C57BL/6 mice showed that APs are eightfold more abundant in SAT than VAT [72, 73].

**Table:** 

Adipogenesis and subsequent AT expansion require appropriate plasticity ensured by efficient remodelling of vasculature and ECM, both processes orchestrated by angiogenic/growth factors and ECM proteases. These processes are also influenced by the anatomical localisation and differentiation capacity of the precursor pools of the different AT depots	

## Difference in AT plasticity between depots in obesity

### WAT expandability: hypertrophy vs hyperplasia in different fat depots

**Fig. 2:**
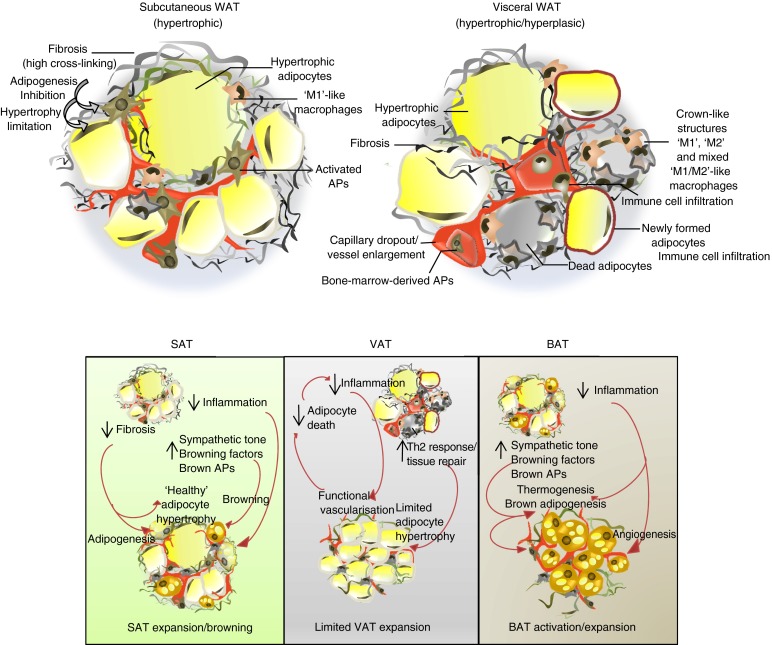
SAT and VAT pathological remodelling in obesity and potential strategies. (**a**) WAT undergoes cellular and structural remodelling in obesity, characterised by the following: (1) adipocyte hypertrophy associated with production of inflammatory factors (VAT > SAT); (2) accumulation of immune cells such as macrophages organised around dead adipocytes (VAT > SAT); (3) decreased capillary density associated with EC dysfunction (i.e. activation, inflammation and senescence) (VAT > SAT); (4) activation of fibroblasts and APs (SAT > VAT) leading to fibrosis deposition and decreased tissue plasticity (SAT > VAT). (**b**) Differential strategies between WAT and BAT depots to prevent obesity-related disorders, targeting tissue plasticity/remodelling and response to sympathetic tone, to promote healthy SAT expansion and browning, conversely with limited VAT expansion and lipotoxic action, and BAT activation and recruitment of APs

Adipocyte hypertrophy is a hallmark of WAT enlargement in obesity and is typically associated with metabolic alterations and increased risk of developing type 2 diabetes, independently from total fat mass. In humans, adipocyte size is positively correlated with glucose intolerance and hyperinsulinaemia [74]. Moreover, inflammation and susceptibility to cell death are both increased in adipose depots with larger adipocytes [75]. Given the differences in adipocyte size/AP pool between SAT and VAT, it is not unexpected that plasticity is also differently affected, particularly when stressed by positive energy balance (Fig. 2a). Similarly, the percentage of small cells is higher in SAT and omental VAT in non-diabetic individuals than in diabetic obese individuals [76]. Recently, an HFD challenge time course experiment in mice revealed intra-depot differences in immune cell composition in relation to WAT expandability [77]. This study also indicated that gonadal VAT is the primary fat depot that expands during the initial phase of obesity, followed by the SAT and mesenteric VAT. Once the mice had reached a body weight of 40 g, gonadal VAT stopped expanding further, in contrast to SAT and mesenteric VAT. Reaching this maximal expansion coincides with increased adipocyte death rate and formation of crown-like structures, inflammation and tissue dysfunction associated with insulin resistance and liver damage [78]. Similarly, another study has suggested that increased visceral mass predominantly results from adipocyte hypertrophy whereas hyperplasia is predominantly seen in SAT [73]. The resistance to differentiation observed in VAT APs and the fact the cells are more prone to cell death than those from SAT, may explain why hypertrophy preferentially occurs in VAT while SAT expands through hyperplasia as a result of the higher progenitor number and/or activity. Consistently, the number of small, early differentiated adipocytes, isolated from human SAT-derived SVF, correlates positively with subcutaneous adiposity (particularly in the femoral SAT), and negatively with VAT accumulation [79]. These data indicate that the abundance of adipocytes/APs in the SAT depots is an important determinant of SAT expandability and functionality. MSCs and pre-adipocytes with proliferative and adipogenic capacity in adult WAT have recently been observed. For instance, while ^14^C birth-dating experiments suggest that the number of adipocytes in SAT is relatively fixed in adulthood independently of BMI, there is now evidence for AP proliferation in human obesity [80]. Specifically, the number of adipocytes is higher in obese than in lean individuals, even after severe weight loss, indicating that increased adipocyte formation in obesity has lifelong effects on AT homeostasis and WAT mass. In mice, an HFD increases adipogenesis in SAT/VAT of young animals but only in VAT of adults. Thus, a reduction in self-renewing division primarily in SAT might explain this phenomenon and suggests that metabolic disease ensues due to a primary failure of SAT plasticity [81]. However, it has been demonstrated recently that increased VAT mass in obese humans is primarily determined by adipocyte number rather than adipocyte hypertrophy [82]. Two independent studies using cell lineage tracing supported human data highlighting higher hyperplasic capacity in VAT compared with SAT during the development of obesity [2, 72]. In the murine model ‘AdipoChaser’, Wang et al showed that the main contributor to tissue expansion is hypertrophy during the first month of an HFD [2]. After prolonged exposure (i.e. <1 month) to HFD, a wave of adipogenesis is initiated preferentially in epididymal AT, whereas only negligible levels of adipogenesis occur in SAT depots. Similarly, Jeffery et al observed increased formation of adipocytes exclusively in VAT after 8 weeks of HFD, using the adiponectin-CreER;mT/mG mouse model to track newly formed adipocytes [72]. This was associated with increased proliferation of APs after the first week on HFD in VAT, but not in SAT, according to BrdU analysis. Timing differences between these studies could have contributed to these discrepancies considering that BrdU analysis of the study involving the AdipoChaser mouse on mice fed HFD was for an extended period of 12 weeks in comparison with the adiponectin-CreER;mT/mG mouse. Taken together, this data suggests that despite characteristically increased cellular proliferation in SAT, adipogenesis may be restricted to VAT at the onset of diet-induced obesity (Fig. 2a).

### Origin of newly formed adipocytes in obesity

In a study in mice, an 8 week HFD led to formation of new adipocytes in VAT, associated with activation and proliferation of Lin^−^Sca1^+^CD29^+^CD34^+^ (CD24^+^ and CD24^−^) adipose progenitors through the PI3K–Akt2 dependent pathway [72]. Interestingly, adipogenesis in response to the HFD did not require Akt2 suggesting a distinct molecular mechanism in obesogenic adipogenesis. This study also showed that APs begin to proliferate only 1 week after the start of the HFD suggesting that AP activation and proliferation in obesity occur before signals produced in response to hypertrophic adipocytes that have reached their maximal storage capacity, at least in the visceral depot. Moreover, the process of forming the mouse fat pad during development can be very different from the route by which new adipocytes are recruited in response to overnutrition.

A subset of bone-marrow-derived circulating progenitors can contribute to adipogenesis as suggested by the small population (2–7%) of green fluorescent protein-expressing (GFP^+^) adipocytes resulting from transplantation of (GFP^+^) bone-marrow-derived cells into mice [83]. This adipogenic process could be optimised (up to 8–25%) in the presence of pro-adipogenic compounds or an HFD. Lineage analysis using LysMcreROSAflox/STOP mice in which LacZ expression is restricted to the myeloid lineage revealed the presence of labelled mature adipocytes, suggesting that bone-marrow-derived adipocytes arise from myeloid progenitor cells [84]. A recent study of 65 individuals who underwent allogeneic bone marrow or peripheral blood stem cell transplantation showed, by taking advantage of genomic differences inherent to donor and recipient cells and performing both bulk and single-cell analyses, that ∼10% of bone marrow serves as a reservoir for AP in SAT, a contribution that increases up to 2.5-fold in obese individuals [85]. However, the haematopoietic origin of adipocytes remains controversial since another lineage-tracing study using a *Vav1*-Cre; R26R-*mTmG* knock-in model, where *Vav1* is a proto-oncogene expressed in the haematopoietic and lymphoid systems, revealed that adipocyte precursors and mature adipocytes from the different WAT depots were negative for the fluorescence [34].

**Table:** 

Hypertrophy preferentially occurs in VAT, while hyperplasia is more characteristic of SAT expansion due to higher progenitor number and/or activity in both human and rodents. However, in chronic states of obesity it is common that SAT adipogenesis is impaired while VAT still expands and contributes to metabolic alterations. Although still controversial, some studies have reported the contribution of haematopoietic precursors to newly formed adipocytes in obesity	

## Pathological regulation of adipogenesis

### Impaired adipogenesis in obesity

Failure of molecular effectors of lipid metabolism could at least partly explain dysregulated lipid storage and mobilisation. In obesity, expression of adipogenic genes (i.e. *PPARγ2*, *FABP4*, *FAS*) is decreased and this is further exacerbated in insulin resistance and type 2 diabetes [86]. Among the different studies highlighting the impairment of adipocyte differentiation in obesity one showed a reduced proportion of committed pre-adipocytes in SVF cells from SAT in obese individuals [87]. Interestingly, low SAT adipogenic rates are strongly associated with visceral obesity, omental adipocyte hypertrophy and metabolic dysregulation, independent of BMI [88]. Also of interest, SAT pre-adipocytes express high levels of mitogen-activated protein 4 kinase 4 (MAP4K4), a kinase induced by TNFα which is known to inhibit PPARγ and subsequent adipogenesis [89].

### Adipogenesis and fibro-inflammation

The effect inflammation has of inhibiting adipogenesis is relatively well characterised [90]. Chronic production of inflammatory factors in obesity results from infiltration and accumulation of immune cells. Among them, pro-inflammatory macrophages are recognised as main effectors, impairing adipogenesis in WAT [91]. The role of immune cells in mediating impairment of brite adipogenesis has also been reported [44]. In fact, inflammation also occurs in BAT from obese mice [92] and inflammatory factors produced by macrophages may inhibit brown adipogenesis [93].

The first wave of WAT accumulation of macrophages during early phases of obesity is essential for healthy tissue expansion and remodelling [94] and if these macrophages become inflammatory they disrupt ECM homeostasis, particularly when the inflammatory insult is sustained, leading to fibrosis deposition. Several studies have reported that the overexpression of ECM components observed in AT from obese individuals and genetically/nutritionally induced obese mice, is linked to metabolic dysfunction causing insulin resistance and liver damage [5, 95]. In addition, fibrosis has a direct negative influence on AT tissue expansion in obesity through impairment of adipogenesis. Several pro-fibrotic factors have been shown to impair human pre-adipocyte differentiation. TGF-β and related members such as activin A are induced in obesity and negatively regulate adipogenesis.

PDGFRα^+^ APs have also been suggested to promote WAT fibrosis [96]. The platelet-derived growth factor (PDGF) is an important profibrotic signal that binds the receptor tyrosine kinases PDGFRα and PDGFRβ [97]. A *Nes*-Cre strategy used to lineage trace pericytes and adventitial cells in WAT showed that despite little contribution of *Nes*-Cre/Tomato^+^ cells to WAT development in young mice, an HFD challenge significantly increases recruitment of PDGFRα^+^ cells [96]. Moreover, in vitro these cells were able to differentiate into adipocytes. However, in vivo activation of PDGFRα signalling causes fibrosis [96]. The importance of PDGFRα signalling in obese WAT fibrosis remains to be tested, but it is tempting to speculate that PDGFRα activation could cause cell-autonomous fibrosis by perturbation of progenitor function.

### Fibrosis limits AT expansion

Fibrosis may also play a direct central role in the expandability of WAT by physically limiting adipocyte hypertrophy [95] (Fig. 2a). In obese mice, WAT fibrosis precedes development of other metabolic complications such as liver damage [78]. Other studies, essentially based on mouse models deficient for ECM or related proteins, support this hypothesis. For instance, increased type VI collagen deposits can be seen in SAT of obese individuals in association with insulin resistance [98, 99]. Genetic ablation of collagens or remodelling enzymes profoundly affects adipocyte size and leads to metabolic consequences. Adipocyte hypertrophy in the absence of fibrotic deposits and inflammation develops in *ob/ob* mice lacking collagen VI in WAT [100]. Despite their severe obesity, these mice are protected from metabolic complications. This suggests that in addition to limiting adipocyte hypertrophy, fibrosis might also impair adipocyte functionality [95, 101].

If accumulation of pericellular fibrosis in SAT is deleterious, there is some recent evidence showing that the fibrotic process in VAT of obese mice limits tissue expansion and related metabolic disorders [102]. In particular, this study revealed that HFD-fed mice with *Irf5* deletion in macrophages display WAT remodelling with accumulation of non-inflammatory macrophages (i.e. involved in ECM remodelling) in VAT leading to fibrosis deposition and limitation of tissue expansion. However, in this case these changes were associated with improved insulin sensitivity.

Given that the amount and activity of BAT also decreases with excess nutrients and fibro-inflammation, fibrotic BAT may exacerbate the development of obesity/complications. Recent evidence also suggests that inflammation and fibrosis negatively affect BAT functions highlighting the role of some molecular candidates involved in vasculature (e.g. VEGF) and ECM turnover, such as TGF-β, endotrophin and microfibril-associated glycoprotein-1 [103–105].

**Table:** 

Adipogenesis is impaired in obesity as a result of a chronic fibro-inflammatory environment where the increase of cytokines and ECM proteins disrupts AP differentiation and promotes activation of fibrotic signalling. In addition, fibrosis mechanically limits tissue plasticity, contributing to metabolic alterations	

## Concluding remarks and future perspectives

Bearing in mind interspecies differences, we suggest that the pathological effects of obesity and related metabolic complications maybe ameliorated by the following: (1) improving the nutrient storage capacity of SAT and simultaneously limiting the storage capacity of VAT by targeting the AT fibro-inflammatory environment; (2) increasing the recruitment capability of WAT precursors and, as a consequence, WAT’s expandability potential and/or (3) increasing BAT mass/activity and SAT browning/beige adipocyte recruitment (Fig. 2b). These strategies will allow the maximisation of the energy-dissipating potential of thermogenic brown and beige adipocytes. In our opinion, this achievement will only be possible with a better understanding of the developmental origins, as well as molecular and physiological nature and plasticity, of adipocytes forming different AT depots in humans during the onset of obesity. This will provide a rationale for translational strategies to improve WAT expandability and brown/beige cell recruitment and activation, moving from rodent models to a clinical context and later on success.

## Abbreviations

AP: Adipocyte progenitor
AT: Adipose tissue
BAT: Brown adipose tissue
BMP: Bone morphogenetic protein
CD: Cluster of differentiation
Cdh5: Cadherin-5
C/EBP: CCAAT/enhancer-binding protein
EC: Endothelial cell
ECM: Extracellular matrix
FABP4: Fatty acid binding protein 4
FGF-2: Fibroblast growth factor 2
FOXC2: Forkhead box C2
GFP: Green fluorescent protein
HFD: High-fat diet
MAP4K4: Mitogen-activated protein 4 kinase 4
MMP: Metalloproteases
MSC: Mesenchymal stem cell
MYF5: Myogenic factor 5
NC: Neural crest
PAX3/7: Paired Box 3/7
PDGF: Platelet-derived growth factor
PDGFRα/β: Platelet-derived growth factor receptor α/β
PI3K: Phosphoinositide 3-kinase
PPARγ: Peroxisome proliferator-activated receptor γ
SAT: Subcutaneous adipose tissue
SREBP1: Sterol regulatory element binding protein 1
SVF: Stromal vascular fraction
TZD: Thiazolidinedione
UCP-1: Uncoupling protein-1
VAT: Visceral adipose tissue
VEGF: Vascular endothelial growth factor
WAT: White adipose tissue
Wt1: Wilms’ tumour 1
ZFP423: Zinc finger protein 423
